# High-Risk Human Papillomavirus (HPV) Status in Colposcopically Diagnosed Low- and High-Grade Cervical Lesions: A Cross-Sectional Study From a Tertiary Care Center in South India

**DOI:** 10.7759/cureus.94723

**Published:** 2025-10-16

**Authors:** Jalagam Naga Revathi, Sornam MS, Preethika A

**Affiliations:** 1 Obstetrics and Gynaecology, Sree Balaji Medical College and Hospital, Chennai, IND

**Keywords:** cervical cancer screening, cervical intraepithelial neoplasia, colposcopy, hpv dna, human papillomavirus virus

## Abstract

Background

Persistent infection with high-risk human papillomavirus (HPV) is the central etiological factor in the development of cervical intraepithelial neoplasia and cervical cancer. While cytology-based screening has been the traditional approach, HPV DNA testing is emerging as a more sensitive modality, especially in resource-constrained settings. This study aimed to determine the prevalence of HPV DNA positivity in women with low-grade (LSIL) and high-grade (HSIL) cervical lesions as identified by colposcopy.

Methods

This was a cross-sectional observational study conducted over 18 months (January 2023 to June 2025) in the Department of Obstetrics and Gynaecology at a tertiary care hospital (Sree Balaji Medical College and Hospital) in South India. Women aged 25-55 years with abnormal cervical findings underwent colposcopic evaluation. Lesions were categorized as LSIL or HSIL based on colposcopic impressions. Cervical samples were subjected to high-risk HPV DNA testing using polymerase chain reaction (PCR). The primary outcome was HPV positivity across lesion grades.

Results

Among 60 participants, 12 (20.0%) were primigravida, 11 (18.3%) were second gravida, and 37 (61.7%) were gravida three or above. Nulliparity was observed in 12 (20.0%) women, while 35 (58.3%) had ≥2 deliveries. Irregular menstrual cycles were reported by 23 (38.3%), postcoital bleeding by 16 (26.7%), and abnormal vaginal discharge by 18 (30.0%). Colposcopy revealed LSIL in 46 (76.7%) and HSIL in 14 (23.3%). HPV DNA was detected in 42 (70.0%) participants, with all HSIL cases [14 (100.0%)] and 28 (60.9%) of LSIL cases testing positive, indicating a significant association between HPV positivity and lesion severity (p = 0.03).

Conclusion

HPV DNA testing demonstrated high detection rates, particularly among women with HSIL, reinforcing its utility as a triage tool in cervical cancer prevention. Integration of HPV testing with colposcopy can enhance early identification and risk stratification of cervical lesions, especially in settings where cytology-based screening is inconsistent.

## Introduction

Cervical cancer continues to be one of the most significant public health challenges in India, where the burden remains disproportionately high despite substantial progress in understanding its etiopathogenesis. The causal link between persistent high-risk human papillomavirus (HPV) infection and cervical cancer is now well established, which has driven the development and implementation of HPV vaccination programs worldwide. In India, initial efforts to introduce HPV vaccination were demonstrated through projects led by Basu and Sankaranarayanan, who highlighted the feasibility, safety, and challenges of integrating vaccination into the existing health system [1]. These pilot programs provided crucial insights into community acceptance, logistical considerations, and the practical difficulties of introducing a preventive strategy on a national scale. Such findings remain highly relevant as India moves toward expanding HPV vaccination coverage.

Globally, cervical cancer ranks among the leading causes of cancer-related morbidity and mortality among women, particularly in low- and middle-income countries (LMICs). According to GLOBOCAN 2022, there were approximately 662,000 new cases and 348,000 deaths worldwide, making cervical cancer the fourth most common cancer in women [2]. India alone accounted for nearly one-quarter of these deaths, underscoring the urgent need for improved preventive strategies and diagnostic interventions. This alarming contribution from India reflects not only the vast population at risk but also the systemic barriers to implementing effective screening and vaccination programs. Consequently, research on the epidemiological and virological characteristics of HPV infection in precancerous cervical lesions has become critical, particularly in resource-limited settings where cervical cancer prevention efforts are still evolving.

The virological profile of cervical cancer is dominated by high-risk HPV genotypes, with HPV-16 and HPV-18 recognized as the most oncogenic. A landmark study by de Sanjose et al. attributed more than 70% of invasive cervical cancers worldwide to these two types [3]. Importantly, their study also emphasized the genotype-specific differences in HPV attribution across histological subtypes, which has significant implications for vaccine design and regional adaptation of prevention strategies. Given that HPV infection is a necessary but not sufficient cause of cervical neoplasia, it is imperative to study its prevalence and genotype distribution across various grades of cervical intraepithelial neoplasia (CIN). Such data are especially important in understanding the risk stratification of low-grade squamous intraepithelial lesions (LSIL) versus high-grade squamous intraepithelial lesions (HSIL).

The progression of cervical cancer has been extensively studied and is widely described as a multistep process, beginning with persistent HPV infection, advancing through CIN, and eventually culminating in invasive carcinoma. Waggoner described this natural history as one shaped by the interplay of viral, host, and environmental factors, with a long latency period between infection and the development of high-grade lesions [4]. This prolonged window offers substantial opportunities for intervention, including screening, early diagnosis, and timely treatment. In LMICs, where access to advanced treatments may be limited, optimizing such preventive opportunities is of paramount importance.

India’s cervical cancer control strategies have shifted significantly over the years, with a growing emphasis on HPV testing as a primary screening tool. Sankaranarayanan et al. demonstrated that a single round of HPV DNA testing reduced both incidence and mortality from cervical cancer more effectively than either cytology or visual inspection with acetic acid (VIA) in rural Indian women [5]. Their findings provided robust evidence that HPV DNA testing is a highly sensitive method, offering clear advantages in resource-constrained settings where cytology-based screening may not be feasible due to limited infrastructure and manpower.

The causal role of HPV in cervical carcinogenesis has been firmly established through epidemiological, molecular, and clinical studies. Bosch et al. presented compelling evidence that HPV fulfills multiple Bradford Hill criteria for causation, including temporality, strength of association, dose-response, and biological plausibility [6]. These findings consolidated decades of research and laid the foundation for incorporating HPV testing into standard screening protocols worldwide.

From a molecular standpoint, HPV infection begins when the virus enters the basal epithelial cells of the cervix, often through microabrasions. Doorbar et al. described the viral life cycle in detail, particularly the oncogenic functions of the E6 and E7 proteins, which disrupt the tumor suppressors p53 and Rb, respectively [7]. These disruptions promote uncontrolled cell proliferation and prevent apoptosis, facilitating the progression toward neoplastic transformation. Persistent infection with high-risk HPV types, combined with viral integration into the host genome, is strongly associated with HSIL and invasive carcinoma, highlighting the need for HPV typing in clinical practice.

The genotype-specific prevalence of HPV varies with lesion grade. Clifford et al. reported that HPV-16 is consistently associated with HSIL and carcinoma in situ, while other types such as HPV-31 and HPV-33 are more frequently detected in LSIL [8]. Such distinctions are clinically meaningful, as they inform both vaccine development and risk-based screening algorithms. Moreover, with the rollout of HPV vaccination programs, ongoing surveillance of genotype distribution is essential to monitor potential shifts in HPV epidemiology due to type replacement.

Recognizing the global burden, the World Health Organization (WHO) launched a call to eliminate cervical cancer as a public health problem. Arbyn et al. emphasized that despite declining incidence rates in some high-income countries, cervical cancer continues to impose a heavy burden in LMICs [9]. The WHO set ambitious elimination targets in 2018: 90% of girls vaccinated against HPV by age 15, 70% of women screened with a high-performance test by ages 35 and 45, and 90% of women with precancer or cancer receiving appropriate treatment. Achieving these goals will require robust regional data, targeted interventions, and sustained political commitment.

Data from the Global Cancer Observatory (GCO), maintained by the International Agency for Research on Cancer (IARC), reinforce the scale of the challenge. The 2020 GCO update reported that South Asia, particularly India, continues to face some of the world’s highest age-standardized incidence and mortality rates for cervical cancer [10]. These statistics underscore persistent gaps in prevention, especially among socioeconomically disadvantaged women who remain unvaccinated and unscreened.

Against this backdrop, the present study was designed to evaluate the HPV DNA status in colposcopically identified high-grade and low-grade cervical lesions. By analyzing the prevalence and distribution of HPV positivity across lesion grades, this study aims to generate data that will inform triage strategies, strengthen early detection, and contribute to the ongoing evaluation of HPV vaccination programs in India. Region-specific insights such as these are indispensable for refining screening algorithms and aligning local practices with global cervical cancer elimination initiatives. This study aimed to determine the proportion of high-risk HPV infection among women diagnosed with HSIL and LSIL and also to compare the prevalence of HPV infection between high-grade and low-grade lesion categories, thereby assessing the potential link between HPV genotype presence and lesion severity.

## Materials and methods

Study design

This research was conducted as a hospital-based observational study within the Department of Obstetrics and Gynaecology at a tertiary care teaching hospital (Sree Balaji Medical College and Hospital) in South India. The study was designed with the primary aim of assessing the association between human papillomavirus (HPV) infection and cervical intraepithelial lesions diagnosed through colposcopic evaluation. While the initial approach was cross-sectional, capturing lesion status and HPV infection at baseline, the study was also structured as a prospective observational investigation, allowing for methodical recruitment and systematic laboratory confirmation. The duration of the study was 18 months, spanning from January 2023 to June 2025, ensuring that the recruitment period was sufficient to capture a representative sample of women attending the hospital’s outpatient clinics. The design enabled the researchers to observe a wide range of patients presenting with abnormal cytology, suspicious screening results, or persistent gynecological symptoms, thus maximizing the validity and generalizability of the findings.

Study setting

The study was carried out at a tertiary care center that caters to both urban and semi-urban populations, offering comprehensive outpatient and inpatient gynecological services. The hospital functions not only as a primary referral unit for smaller healthcare facilities but also as a center where women often present on their own with gynecological complaints or for routine screening. This diversity of the catchment population provided a mix of socio-demographic and clinical profiles, thereby strengthening the applicability of the findings to similar healthcare contexts across the country.

Study population

The study population consisted of women referred for colposcopic evaluation due to abnormal cervical cytology, the presence of persistent clinical symptoms, or suspicious findings detected during routine gynecological screening. Women typically presented with abnormal vaginal discharge, postcoital bleeding, intermenstrual spotting, or nonspecific pelvic symptoms, while some were identified through regular preventive check-ups. Those who underwent colposcopy and were subsequently diagnosed with either low-grade squamous intraepithelial lesion (LSIL) or high-grade squamous intraepithelial lesion (HSIL) formed the analytic cohort for HPV testing and further evaluation. By focusing on this subset, the study specifically targeted women most likely to benefit from HPV testing as part of their clinical assessment.

Eligibility criteria

Eligibility for participation was defined by carefully framed inclusion and exclusion criteria. Women were eligible for the study if they were between 25 and 45 years of age, had been diagnosed with either LSIL or HSIL on colposcopic assessment, and had provided informed written consent to undergo HPV testing and further gynecological evaluation. Exclusion criteria comprised postmenopausal women, those not leading an active sexual life, patients diagnosed with invasive cervical carcinoma, and women with normal colposcopic findings. In addition, patients with gross cervicitis, acute pelvic inflammatory disease, or active vaginal infections were excluded in order to avoid confounding factors that might influence HPV testing or colposcopic findings. Finally, women who were unwilling to undergo colposcopy or HPV DNA testing were also excluded. These criteria ensured that the study population was relatively homogenous, with a focus on women at risk for or already presenting with precancerous cervical lesions.

Sample size and sampling method

A purposive sampling strategy was adopted, wherein women fulfilling the eligibility criteria were consecutively recruited from the outpatient department during the study period. The sample size was calculated using conventional formulae for prevalence studies, taking into account the expected prevalence of HPV infection among women with cervical intraepithelial lesions, a 95% confidence interval, and an acceptable margin of error. Based on these calculations and the outpatient load of the hospital, the required sample size was determined to be 60 women. This number was considered adequate to detect meaningful differences in HPV prevalence between women with LSIL and HSIL.

Data collection

Once enrolled, participants underwent a structured interview and clinical evaluation. Data were collected using a predesigned and pretested proforma that had been refined through pilot testing to ensure clarity and comprehensiveness. The information collected included socio-demographic details such as age, parity, educational status, and socioeconomic background, which was classified according to the modified Kuppuswamy’s scale. Obstetric and gynecological history, including menstrual profile, contraceptive practices, history of sterilization, and the presence of symptoms such as postcoital bleeding or abnormal vaginal discharge, was recorded in detail. Medical history was elicited with particular attention to comorbidities such as diabetes mellitus and hypertension, while family history was probed for genital tract malignancies. Sexual and reproductive histories were also recorded, given their potential relevance to HPV infection risk. A thorough pelvic examination was carried out to exclude acute infections or abnormalities that could interfere with colposcopic assessment.

Colposcopic examination

Colposcopic evaluation was performed for all eligible women using a high-resolution video colposcope under adequate illumination and magnification. The procedure followed a standardized protocol that began with the application of normal saline to the cervix to enhance visualization of the epithelial surface and vascular patterns. This was followed by the application of 3-5% acetic acid, which produced acetowhitening of abnormal areas and made visible specific features such as punctation, mosaicism, and atypical vascular changes. Lugol’s iodine solution was then applied to differentiate glycogenated from non-glycogenated epithelium. Colposcopic findings were classified according to the International Federation for Cervical Pathology and Colposcopy (IFCPC) nomenclature introduced in 2011. Based on the impressions recorded, lesions were categorized into LSIL or HSIL. In cases where lesions were visible, directed cervical punch biopsies were obtained from the most suspicious areas for histopathological confirmation, ensuring that diagnostic accuracy was maintained.

HPV testing

Women diagnosed with LSIL or HSIL on colposcopy subsequently underwent HPV DNA testing. Cervical samples were obtained using a cytobrush and transferred into a liquid-based cytology medium. The samples were preserved and transported under strict cold-chain conditions to the molecular diagnostic laboratory. The Hybrid Capture 2 (HC2) assay was employed for HPV detection, as it is a highly sensitive and validated nucleic acid hybridization test with signal amplification that can identify 13 high-risk HPV genotypes-HPV 16, 18, 31, 33, 35, 39, 45, 51, 52, 56, 58, 59, and 68. Among these, HPV 16 and 18 are responsible for approximately 70% of cervical cancers worldwide, while the remaining genotypes are associated with varying degrees of oncogenic potential, contributing to the residual 30% of cases.

The testing process involved denaturation of viral DNA into a single-stranded form, hybridization with complementary RNA probes, capture of DNA-RNA hybrids on microplates coated with specific antibodies, signal amplification through conjugated alkaline phosphatase, and chemiluminescent detection. Light emission was measured in relative light units, and results equal to or above the predefined threshold were considered positive for high-risk HPV infection. The HC2 assay was preferred over PCR-based genotyping due to its standardized protocol, reproducibility, and suitability for high-throughput screening in resource-limited settings. Moreover, it provides clinically actionable information on the presence of any high-risk HPV infection, which is more relevant for triaging and management than genotype-specific identification in this study context. All laboratory staff were blinded to clinical and colposcopic findings to eliminate observer bias.

Classification of participants

Following HPV testing, participants were classified into four groups based on the combination of lesion grade and HPV status. Women with LSIL who tested positive for HPV were grouped separately from those with LSIL who were HPV negative. Similarly, women with HSIL were categorized according to whether their HPV test results were positive or negative. This classification enabled structured analysis of the relationship between HPV positivity and lesion severity, and facilitated the identification of infection patterns across groups.

Outcome measures

The primary outcome measure of the study was the proportion of HPV positivity among women diagnosed with LSIL and HSIL. Secondary outcomes included the association between HPV status and demographic factors such as age, education, parity, and socioeconomic class, as well as clinical factors including sterilization, menstrual pattern, and presenting symptoms like postcoital bleeding or vaginal discharge.

Ethical considerations

The study protocol was reviewed and approved by the Institutional Human Ethics Committee of the institution (002/SBMCH/IHEC/2023/2037). Written informed consent was obtained from all participants in their preferred language. Ethical principles outlined in the Declaration of Helsinki were strictly followed. Participants were informed that their involvement was voluntary and that they could withdraw at any stage without compromising their clinical care. No financial incentives were provided, and no experimental drugs or interventions were administered as part of the study.

Statistical analysis

Data were double-entered into Microsoft Excel 2019 (Microsoft Corp., Redmond, WA, USA) to minimize errors and subsequently exported to SPSS version 26.0 (IBM Corp., Armonk, NY, USA) for analysis. Descriptive statistics, including means and standard deviations for continuous variables and frequencies and percentages for categorical variables, were used to summarize baseline characteristics. Inferential statistics were applied to test associations between HPV status and lesion grade, as well as between HPV status and other demographic or clinical factors. The Chi-square test was employed where appropriate. A p-value of less than 0.05 was considered statistically significant. This analytical framework was chosen to ensure both robustness and reproducibility of the study findings.

## Results

The socio-demographic profile of the study participants (n=60) is summarized in Table 1. The majority of women were aged between 36-40 years [18 (30.0%)], followed by those aged 41-45 years [16 (26.7%)], while 14 (23.3%) participants were between 31-35 years and 12 (20.0%) were in the 25-30 year age group. Educational attainment showed that 20 (33.3%) women had completed secondary education and an equal proportion, 20 (33.3%), were graduates, whereas 10 (16.7%) were illiterate and another 10 (16.7%) had only primary education. In terms of socioeconomic status, 21 (35.0%) belonged to the lower socioeconomic class, 16 (26.7%) to the lower-middle class, 9 (15.0%) to the upper-middle class, while 7 (11.7%) each were in the upper-lower and upper socioeconomic categories.

**Table 1:** Socio-demographic characteristics of the study participants (n=60) *Modified BG Prasad Scale [11].

Variable	Frequency (n)	Percentage (%)
Age category		
Age 25–30	12	20.0
Age 31–35	14	23.3
Age 36–40	18	30.0
Age 41–45	16	26.7
Education		
Illiterate	10	16.7
Primary Education	10	16.7
Secondary Education	20	33.3
Graduate	20	33.3
Socioeconomic Status*		
Lower Socioeconomic Status	21	35.0
Lower-Middle Socioeconomic	16	26.7
Upper-Lower Socioeconomic	7	11.7
Upper-Middle Socioeconomic	9	15.0
Upper Socioeconomic	7	11.7
Marital Status		
Divorced	2	3.3
Married	36	60
Single	19	31.7

The reproductive and clinical characteristics of the study participants in Table 2 showed that 12 women (20.0%) were primigravida (G1), 11 (18.33%) were second gravida (G2), and the majority, 37 (61.67%), were gravida three or above. In terms of parity, 12 women (20.0%) were nulliparous (P0), 13 (21.67%) were primiparous (P1), and 35 (58.33%) had two or more deliveries (P2 and above). Regarding sterilization, most participants, 52 (86.67%), were not sterilized, while only 8 (13.33%) had undergone sterilization. Menstrual cycle analysis revealed that 37 women (61.67%) reported regular cycles, whereas 23 (38.33%) experienced irregular cycles. Postcoital bleeding was reported by 16 participants (26.67%), while 44 (73.33%) denied such symptoms. Abnormal vaginal discharge was present in 18 women (30.0%) and absent in 42 women (70.0%).

**Table 2:** Distribution of obstetrics and menstrual characteristics among the study participants (n=60)

Variables	Count	%
Gravida		
G1	12	20
G2	11	18.33
G3 and above	37	41.67
Para		
P0	12	20
P1	13	21.67
P2 and above	35	58.33
Sterilization Status		
Not sterilized	52	86.67
Sterilized	8	13.33
Menstrual Cycle Regularity		
Irregular	23	38.33
Regular	37	61.67
Postcoital Bleeding		
Absent	44	73.33
Present	16	26.67
Abnormal Vaginal Discharge		
Absent	42	70
Present	18	30

Colposcopic evaluation of the study participants revealed that high-grade squamous intraepithelial lesion (HSIL) was detected in 14 women, accounting for 23.3% of the cases, while low-grade squamous intraepithelial lesion (LSIL) was more common, observed in 46 women, representing 76.7% of the total. Among the study participants, human papillomavirus (HPV) testing revealed that 42 women were HPV positive, accounting for 70% of the cases, while 18 women were HPV negative, representing 30%. The colposcopic findings among the study participants (n=60) revealed that 14 women (30.0%) were diagnosed with high-grade squamous intraepithelial lesion (HSIL), while the majority, 46 women (70.0%), had low-grade squamous intraepithelial lesion (LSIL). All HSIL cases (100.0%, 95% CI 78.2% - 100%) tested positive for high-risk HPV, whereas none were HPV negative. Among the LSIL group, 28 women (60.9%, 46.6% - 73.4%) were HPV positive, while 18 (39.1%, 22.9% - 51.3%) were HPV negative. Overall, HPV DNA was detected in 42 participants (70.0%) and absent in 18 participants (30.0%), indicating a strong association (p-value = 0.03) between HPV positivity and lesion severity, with universal positivity in HSIL cases and a comparatively lower prevalence in LSIL cases (Table 3).

**Table 3:** Association between colposcopy findings and HPV test results *p-value < 0.05 is statistically significant

Colposcopic Diagnosis	HPV Negative n (%)	HPV Positive n (%)	Chi-square	p-value
HSIL	0 (0.0)	14 (100.0)	7.07	0.03*
LSIL	18 (40.0)	28 (60.0)		

## Discussion

The current study aimed to determine the HPV status in HSIL and LSIL cervical lesions using colposcopic diagnosis and HPV DNA testing in a tertiary care setting. Among the 60 women enrolled, 14 were diagnosed with HSIL and 46 with LSIL based on colposcopic assessment. The findings demonstrated that HPV DNA was detected in 100% (14/14) of HSIL cases and 60.9% (28/46) of LSIL cases. This aligns with the understanding that high-grade lesions are more strongly associated with persistent high-risk HPV infection compared to low-grade lesions, and reinforces the importance of accurate screening modalities in resource-limited settings like India.

Colposcopy remains a crucial diagnostic tool in the assessment of cervical intraepithelial neoplasia. Sellors and Sankaranarayanan have emphasized the importance of colposcopic accuracy in identifying lesion severity, especially in low-resource settings where histopathological confirmation may be delayed or unavailable [12]. In our study, colposcopic assessment correlated well with HPV status, reinforcing its utility as a frontline diagnostic modality. The use of colposcopy enabled real-time assessment of lesion characteristics, providing an effective triage method when combined with HPV testing.

Wright et al., in the ATHENA study, confirmed that HPV DNA testing has higher sensitivity than cytology for detecting high-grade cervical lesions, a finding supported by our results showing complete concordance between HSIL and HPV positivity [13]. Our data suggest that HPV DNA testing may outperform cytology in high-risk populations, and highlight the potential benefit of using molecular methods as a primary screening strategy. Similarly, Denny et al. emphasized the role of HPV testing as a primary screening method in developing countries, where cytological services may be inconsistent or unavailable [14]. Our study reinforces this recommendation by demonstrating a significant HPV burden in colposcopically evident lesions, especially among high-grade abnormalities.

Munoz et al. provided an epidemiologic classification of high-risk HPV types associated with cervical cancer and concluded that types 16 and 18 accounted for the majority of high-grade lesions globally [15]. Though genotype-specific data were not obtained in our study, the strong correlation between lesion grade and HPV positivity supports the global trend of genotype predominance in severe lesions. Our data emphasize the urgent need to incorporate genotype analysis in future research to better define the regional distribution of high-risk HPV strains in India.

Smith et al. performed a meta-analysis showing that HPV-16 and -18 were found in over 70% of HSIL cases across multiple continents [16]. This parallels our observation of complete HPV positivity in HSIL, suggesting that the burden of oncogenic HPV in high-grade lesions is consistent regardless of regional variations. Furthermore, it reinforces the value of HPV vaccination, which targets these genotypes, in significantly reducing the burden of severe cervical lesions.

Castellsagué et al. highlighted that adenocarcinomas, though less common, were also predominantly HPV-related, especially to type 18 [17]. While our study focused solely on squamous lesions, it indirectly supports the pathogenic significance of HPV across all histological subtypes. A comprehensive cervical cancer prevention strategy must therefore address all variants of HPV-related cervical neoplasia.

Indian studies such as those by Pankaj et al. and Vinodhini et al. have demonstrated varied HPV prevalence in cervical lesions, ranging from 25% in LSIL to nearly 100% in HSIL cases, which closely mirrors our findings [18,19]. These regional studies corroborate our HPV detection rates, suggesting consistent epidemiological patterns across different Indian states. Our study's HPV positivity in LSIL (60.9%) is within the reported national range and supports existing evidence of high HPV prevalence in high-grade disease. Importantly, the moderate HPV positivity in LSIL also highlights the potential for spontaneous clearance in low-grade lesions, emphasizing the need for close follow-up rather than overtreatment.

Origoni et al. reported similar findings where HPV DNA was detected more frequently in higher-grade lesions, reinforcing the diagnostic value of molecular testing [20]. Likewise, Bowden et al. noted regional differences in HPV risk factors, including early sexual activity, multiple partners, and tobacco use, but confirmed the consistent association between HPV positivity and lesion severity [21]. These findings underline the need for targeted screening in high-risk subpopulations. Katki et al. reported a five-year cumulative risk of CIN3+ and cervical cancer in women who tested HPV-positive and Pap-negative, affirming the predictive power of HPV testing even in the absence of cytological abnormalities [22]. This is significant for our findings, as colposcopy and HPV testing together offered better lesion stratification. The combination of a visual and molecular method enhances detection accuracy and risk prediction, particularly valuable in settings with inconsistent cytological services.

Guidelines from ASCO, as stated by Jeronimo et al., advocate for resource-stratified cervical cancer prevention strategies with a strong emphasis on HPV testing in triage and follow-up [23]. Our study findings support this, especially in settings with limited access to cytology-based screening. Integrating HPV testing into national screening guidelines would greatly benefit underserved populations and enable earlier diagnosis of precancerous lesions. Bhatla et al., in their review of cervical cancer in India, emphasized the integration of HPV testing into existing screening protocols to improve early detection and reduce disease burden [24]. This is in concordance with our findings that HPV testing provides valuable information for risk-based triage. Our results provide an evidence base to support policy-level decisions regarding the transition from cytology-based to HPV-based screening in India.

Cuzick et al. summarised European and North American HPV screening trials and concluded that HPV-based screening was more effective than cytology alone in preventing cervical cancer [25]. The present study adds to this global evidence, particularly from an Indian context. By aligning with international findings, our data contribute to the universal applicability of HPV-based strategies in cervical cancer prevention. Finally, zur Hausen’s seminal work on the oncogenic potential of HPV laid the foundation for all subsequent screening, diagnostic, and vaccine strategies in cervical cancer [26]. The complete HPV positivity in high-grade lesions seen in our study strongly validates his hypothesis and its translational application. His identification of HPV as a causative agent revolutionized the field of cervical cancer prevention and remains the cornerstone of all current interventions [27].

Our study had certain limitations that must be acknowledged. First, the sample size was relatively small (n = 60), which may limit the generalizability of the findings across diverse populations. Second, HPV genotyping was not performed, and therefore we could not distinguish between individual high-risk HPV subtypes such as HPV 16 and 18, which are most strongly associated with high-grade cervical lesions. This limited our ability to determine genotype-specific prevalence within the regional cohort. Third, histopathological confirmation of lesion grade was not uniformly available, only 41 out of 60 cases (68.3%) had corresponding biopsy results. The remaining cases were classified based solely on colposcopic impression, which, while widely accepted, is subject to inter-observer variability and may influence diagnostic accuracy. Fourth, the cross-sectional study design precludes assessment of lesion progression or regression over time; longitudinal follow-up would be necessary to understand the natural history of HPV-positive LSIL cases.

Finally, an important limitation pertains to the HPV-DNA testing method itself. Although HPV-DNA testing is highly sensitive for identifying high-risk infections, not all women with cervical lesions or even invasive cervical cancers test positive for HPV DNA. This raises the possibility of false-negative results if HPV-DNA testing were to be introduced as a standalone screening tool. Consequently, exclusive reliance on HPV-based screening could miss a small subset of HPV-independent cervical cancers, underscoring the need to integrate cytology or colposcopic evaluation in national screening programs.

Despite these limitations, our study reinforces the critical role of HPV testing and colposcopy as complementary tools in the detection and triage of cervical intraepithelial lesions, especially in low-resource settings. The high rate of HPV positivity in HSIL underscores the need to prioritize HPV-based screening strategies for early identification of high-risk cases. Incorporating HPV genotyping in future studies would help tailor vaccination and screening programs to regional HPV subtype prevalence. Policy-level integration of HPV DNA testing into national screening guidelines could substantially improve early detection, reduce overtreatment of LSIL, and optimize resource allocation. In the broader context, our findings support the global evidence base advocating HPV vaccination and molecular testing as cornerstones of cervical cancer prevention strategies, with particular relevance for resource-constrained healthcare systems such as India.

## Conclusions

This study underscores the strong association between high-risk HPV DNA positivity and the severity of cervical lesions, with 100% of high-grade squamous intraepithelial lesion (HSIL) cases and 60% of low-grade squamous intraepithelial lesion (LSIL) cases testing positive for HPV. These findings align with international evidence and reaffirm the pivotal role of high-risk HPV infection in cervical carcinogenesis. The markedly higher prevalence of HPV in HSIL highlights the potential utility of HPV DNA testing as a reliable triage tool for prioritising women for colposcopic evaluation and biopsy, particularly in resource-limited settings where cytology-based screening may not be feasible. Given India’s high cervical cancer burden and the recent introduction of affordable indigenous HPV vaccines, the results provide timely evidence to support the policy-level integration of HPV-based molecular screening into the National Programmes and the ongoing National Cervical Cancer Elimination Initiative. Scaling up HPV testing - either as a primary screening method or as a co-test with cytology - could substantially enhance early detection, optimize resource use, and reduce mortality. By elucidating the distribution of HPV positivity across lesion grades, this study contributes to more precise risk stratification and early intervention strategies. Future research with larger cohorts, genotype-specific analysis, and longitudinal follow-up is warranted to validate these findings and further guide India’s evolving cervical cancer screening policy toward elimination goals.
